# Notes of Epidemic and Other Diseases That Have Prevailed in the United States Since Its First Settlement

**Published:** 1850-05

**Authors:** C. A. Lee

**Affiliations:** Prof. of Mat. Med. and Gen. Pathology in the University of Buffalo


BUFFALO MEDICAL JOURNAL

AND

MONTHLY REVIEW.

VOL. 5. MAY, 1850. NO. 12.

### ORIGINAL COMMUNICATIONS.

ART. I.—*Notes of Epidemic and other Diseases that have prevailed in the United States since its first settlement.* By C. A. LEE, M. D., Prof. of Mat. Med. and Gen. Pathology in the University of Buffalo.

It would seem to be the order of Providence, that the population of the globe we inhabit, should be kept at about the same limit it has long since attained; and to this end epidemic diseases of a fatal character, are from time to time permitted to sweep away immense multitudes of the human race. History is full of such records, and our own annals abound in similar melancholy details. It is an interesting, and, at the present time, much agitated question, whether the ravages of pestilential and other diseases may not be prevented by proper sanitary regulations, public and private, and the average life of man be made to correspond with the period assigned him in the holy writ, three score years and ten. If these epidemics were owing to some poisonous effluence from planitary bodies or the bowels of the earth, inimical to all animal life, and under all circumstances injurious, as some contend; or the product of animalculæ or vege table fungi, against which no one can guard, and which may prevail in all places alike, then it were useless to attempt sanatory ameliorations or hygienic safeguards; boards of health may be dismissed, and quarantines neglected, and all may be permitted to follow their own inclinations, live as they please, and wallow in filth, intemperance, and mire, to their heart's content. But such is not our creed. We believe that the world is governed by general laws; that these laws are founded on principles of rectitude and justice—that they may be learned by studying nature, as well as from revelation—that we do understand in some good degree, those connected with the organization and functions of the human body, and that if we obey them, health, happiness, and long life will be the result Life may be cut short by accidents against which we cannot guard—there may be an heredetary taint or vice in the constitution, which is calculated to cut short the existence of individuals—disease may supervene upon unavoidable exposure, hardship, or the use of innutritious or unsuitable food —the hardy may suffer and perish from severe mental shock, or the corroding influence of slow grief—poisons may be imbibed, too virulent for the strongest forces to resist—infantile life may speedily be extinguished from feeble vital force—but notwithstanding all these exceptions, the value of human life may be vastly increased, and will be, when the organic laws of our being, physical, moral, and intellectual, are heeded and obeyed. Till then cholera must decimate our cities and principal towns—infectious fevers must have their victims—malaria must find its hecatombs, contagious exanthems must sweep off multitudes—yellow fever, dysentery, plague must continue their ravages, and man cannot live out half his days, as is actually the fact the world over.

The following notices of epidemics which have prevailed in this country, would be far more valuable, could we in all cases connect them with the causes to which they owed their prevalence. That such causes existed, is self-evident; in some cases they are ascertained, in others surmised.

In the year 1618, a very severe pestilence raged among the Indians of New England, so that Hutchinson states (vol. 1, p. 34, 35,) that 30,000 of the Massachusetts tribe were reduced to 300. It seems to have been a pestilential fever, of a typhoid type, attended with swelling and suppuration of the glands of the axillæ, groin, &c., as in plague, the skin being "exceedingly yellow." Noah Webster thinks it was the yellow fever, but this is too improbable to merit notice. It was probably caused by famine, and living on innutritious diet, such as the bark and roots of trees, and other crude food. The disease prevailed from 1617 to 1623.*

In 1619, 300 of the settlers in Virginia died, chiefly of fevers; and out of 4,170 inhabitants in 1621, there were but 1800 living in 1624. Fevers and scanty means of subsistence were assigned as causes of this great mortality. (Purchus.)

In 1633, a typhoid fever, called by Hubbard "a pestilent fever," swept off 20 of the small colony at Plymouth, and during the same year the In-

*See Purchas, vol. 4, 1778, and 4, 1858; Belknap's Life of Gorges, vol. 1, p. 355; Hayward's Collection, vol. 1, p. 148; Prince's Chron. 124; Magnolia, b. 1, p. 7; Hutchinson, vol. 1, p. 34; Winthrop's Jour. p. 53.

dians of New England were swept off in large numbers by the small pox.*

In 1635, 1800 persons died in Virginia of dysentery. (Winthrop.)

1638 was a very sickly year in New England, and a general fast was ordered on account of the "small pox and fevers." (Winthrop.)

The summer of 1642 was very sickly among the settlers on the Delaware river. The mortality among the settlers from New Haven was sucl as to break up their settlement. (Winthrop, p. 240.) The disease seems to have been a remittent bilious fever.

Great sickness prevailed among the Indians on Martha's Vineyard in in 1645—few escaped. (Neal's History of New England, vol. 1, p. 264.)

In 1647, the first epidemic catarrh or influenza prevailed in America of which we have any record. It is described by Hubbard as attacking all, both colonists and natives, and as beginning with a cold, and in many accompanied with a light fever. Such as bled or used cooling drinks, he says, died, while those who used cordials and tonics, recovered. In Barbadoes and St. Kitts, 5000 or 6000 are represented to have died in each.† The same year a "malignant fever" prevailed in Hartford, and other parts of Connecticut, which was attributed to the excessive heat of the summer.‡

A second epidemic influenza prevailed throughout America in 1655. Hubbard says: "In 1655 there was another faint cough that passed through the whole country of New England, occasioned by some strange distemper or infection of the air. It was so epidemical that few persons escaped. It begun about the end of June. Few were able to visit their friends or prepare the last testimony of respect to any of their relations at a distance." (Hub. manuscript, p. 285—Magnolia v. 3, p 108.)

Trumbull states that there "was a great sickness and mortality throughout New England in 1658. The season was intemperate and the crops light."—(Trum. 6, Hist. p. 244.) In 1659 we find the first mention made of the Cynanche Trachealis (Croup,) in America, (Magnolia 6. 4. p. 156.) The year 1662 was characterized by malignant disease, and the Legislature of Conn. appointed a day of thanksgiving in Oct., for the "abatement of the sickness in the country, and a supply of rain in time of drought.—(Loc. Cit.)

In 1664, we are informed by N. Webster, that the mildew of wheat

---

*Hubbard's MSS., p. 131; Winthrop's Jour. 51, 56, 59, 61.
†Hubbard's MSS,, p. 276.
‡Neal's Hist. of N. E., vol. 1, p. 289; Magnolia, b. 3, p. 67.

commenced in New England, "which has rendered it impossible to cultivate that grain, on the Atlantic coast of the three Eastern States."—(on Epidermics Vol. p. 194.)

The small pox prevailed extensively in Boston in 1666. The summer of 1668 was extremely hot, and malignant diseases (fevers) very generally prevailed. In New York Fever was so fatal, that a fast was appointed in Sept. on that account. The disease was evidently a high grade of bilious autumnal fever—(Neal's Hist. v. 1, p. 367—Magnal, 64, 184.)

In 1677, the small pox was very prevalent and fatal in Charlestown Mass—Mag. 64, p. 189)—and the next year in Boston. For several years at this period, say from 1669 to 1678, the seasons were unfavorable, and the fruits blasted, and malignant diseases prevailed among the people. Our ancestors attributed the bad seasons and the sickness to the irreligion of the times, and their disuse of fasting. A Synod was, on this occasion, convened to investigate the causes of God's judgments, and to propose a plan of reformation.—(Neal's Hist. vol. 2, p. 32—Mag. 65, p. 85—Hutch. vol. 1. 324—Doug. vol. 1, 440—Short. vol. 1.)

The winter of 2683–4, was one of the coldest in N. E. on record, and also very sickly—(Ellicot's Hist., Call. v. 3.)

The year 1683 was also remarkable for general sickness and unusual mortality in Conn., and many towns suffered from excessive rains.—(Trumbull's Hist. p. 383.)

In 1693, Sir Francis Wheeler was sent with a fleet from Eng. to conquer the island of Martinics—while there the seamen and soldiers were seized with "the plague of America," probably yellow Fever, or a high grade of bilious, and three-fourths of them perished. Mather says, (Magnol. vol. 2, p. 91) "the distemper was most like the plague of any thing that had ever been seen in America, whereof three died before the fleet could reach Boston, as I was told by Sir Francis himself, 1300 soldiers out of 2100, and 1800 soldiers out of 2400—(Loc. Cit.) It does not appear that the disease was propagated at all in Boston, on the arrival of the fleet, from Mather's account, although Hutchinson states expressly that it was.

In 1695 a mortal sickness prevailed among the Indians of New England and Canada—(Hutch. vol. 2, 89.) In 1697–8 the Influenza began in Nov., and prevailed over the country in a very severe form, till Feb., being most violent in Jan., when "whole families were sick at once, and whole towns seized at nearly the same time. In the same winter a very fatal typhus fever prevailed in the town of Fairfield, Conn., which was so general that well persons could scarcely be found to tend the sick and bury the dead." Seventy persons were buried in three months out of a population of less than 1000.—(Trumbull.) It also was rife in some parts of New Hampshire, as Dover.

In 1600, a very fatal fever, called "bilious plague," but which, I doubt not, was yellow fever, raged in Charleston, S. C., and in Philadelphia. It broke out about the beginning of August, in Philadelphia, and is called by some writers at the time, the "Barbadoes distemper," and the patients are said to have "vomited and voided blood." The disease raged till the middle of Oct., of which 210 died. The summer is stated to have been the hottest ever known; "persons died at harvest in the field"—150 died Charleston, in a few days, and most of the survivors fled into the country, and we are informed that " the principal officers of government, one half of the members of the Assembly, and multitudes of the citizens fell victims."—(Hist. of S. C., vol. 1, p. 142.) This seems to have been the first record of yellow fever in Philadelphia.

Honest Cotton Mather, in a sermon, preached in Boston on " Lecture Day," Sept. 29th, 1698, says, "the harvest hath once and again grieviously failed. The very course of nature hath been altered among us; a lamentable cry for bread, bread, hath been heard in our streets."—(Magnal, 69, 113.) And in the same sermon he says, " Epidemical diseases have in three years, been once and again upon us"—and it is stated that " Boston lost in one year, 600 or 700 of its people, by one contagious disease"—probably typhus fever.

A malignant small pox raged in Boston in the year 1702, attended in many cases with a scarlet eruption, which was mistaken for scarlet fever. It began in June and was very mild, in Sept. and Oct. it became very fatal, and the Gen. Court sat at Cambridge. The scarlet fever and variola both were very rife and fatal till Feb., when they began to subside.

In this same year, 1702, the yellow fever raged very violently in the city of New York, and was believed to have been imported from St. Thomas. It was called the " *Great Sickness*," and hardly any survived. The Assembly was held, on account, at Jamaica, L. I.—)Smith's Hist. of N. Y., p. 104—Gen. Assem. v. 1, 151.)

In 1709, the troops under Gen. Nicholson, destined for the invasion of Canada, were attacked with bilious intermittent fever and dysentery when encamped at Wood Creek, N. Y., and many perished. Charlevaix accounts for the sickness by saying that the Indians had poisoned the water of the creek, by throwing into it all the skins of beasts they had taken in hunting.

The typhoid pleurisy was very fatal in some parts of Conn., as at Waterbury, in 1712—(Trum. Hist. of Conn. 386.) In 1713, Epidemic measles was rife over many parts of New England. The winter of the year 1717–18, was very cold, the snow very deep, and it was also remarkable for the death of many old people. In 1719, a malignant pleurisy raged in some parts of New England, and great mortality in some parts of Delaware from fevers. Webster states that in the year 1723 a fatal disease, called the "burning ague," prevailed in some parts of Rhode Island, and was particularly fatal near Providence." It did not prevail in large towns, but in villages, and perhaps the clearing of some neighboring swamps might have been one cause of the disease."—(vol, 1, p. 229.)

The summer of 1728 was a remarkably hot one, and the yellow fever was very fatal in Charleston, S. C. (Hist. of S. C. vol. 1, 316.) The measles prevailed epidemically in New England, in 1728, and in many places in Conn., a malignant pleurisy. This latter disease indeed was very rife in the northern parts of the U. S., for many years, Webster says, until 1761—(Epid. vol 1. 231)—The bills of mortality of 1730 and '31 show that the mortality was remarkably great in Phil. and Boston; the excess of deaths in Boston, seems to have been occasioned by the small pox; in Philadelphia the greatest No. of deaths was in March and April. In 1732 the yellow fever again ravaged Charleston S. C.—(Lining. Ed. Ess. vol. 2.) The influenza spread over the whole of N. America in the same year, commencing in Oct., and in the year following it extended over Europe; in this instance, at least, moving from West to East. In May, 1735, we first hear of the "throat distemper," (cynanche maligna,) among children in this country; it ravaged some parts of New Hampshire, and probably the other New England States in a very malignant and fatal form. The prominent symptoms were a "swelled throat, with white or ash colored specks, an efflorescence on the skin, great debility of the whole system, and a tendency to putridity. It first seized a child (at Kingston,) "who died in three days. In about a week afterwards, three children in another family, at a distance of four miles, were successively seized, and all died on the third day. It continued to spread, and of the first forty patients, not one recovered."—(Webster's Epid. vol. 1, p. 233.) This was in May 1735, during a wet and cold season. It appeared at Exeter, N. H , only six miles distant, in the ensuing Aug., and in Boston, 50 miles distant, it broke out in September; although it did not appear in Chester, six miles west of Kingston, until October. It continued its ravages through that year into the next, and gradually traveled southward, almost stripping the country of children. Very few children escaped, for although the disease was very infectious, yet its propagation depended very little on that circumstance. It attacked the young in the most sequestered situations, and without a possible communication with the sick. It was literally the plague among children. Many families lost three and four children—many lost all. (Webster *loc cit.*) Country places suffered more than cities by this disease. It was aggravated by cold and wet weather. Scorbutic people, and those who ate much pork, and of course the poor, suffered the most. It varied greatly in severity in different families. It gradually traveled west, and it was two years before it reached the Hudson River. It continued spreading to the west, until it spread over the whole country. Its ravages, as now, were chiefly confined to children and persons under puberty. For many years afterwards, it broke out in different places, in an epidemic form, but did not spread as at first. Those who recovered of the disease, it is said, were always liable afterwards to sore throats and quinsies, and few or none lived to be old. Some accounts represent the invasion of the disease as very gradual, children appearing to languish for some time previous to an attack. It is also said, that it was not always attended with great prostration of strength, patients often walking about an hour or two before death. (Colden, med. ob. and Eng., vol. 1, 211. Belknap's N. Hamp., vol. 2, 118). During the year 1737, a high grade of bilious fever, was very fatal in Virginia, and a very severe influenza also prevailed over both hemispheres. In 1739, the small pox and dysentery were quite fatal in New York city, and a contagious fever ravaged Charleston, S. C. (Jour. N. Y. Assembly, vol. 1. Fothergill on Sore Throat). In 1740-41, we read of the measles prevailing epidemically in Connecticut, and the yellow fever in Philadelphia and Virginia. The sca latina maligna was very rife in some parts of New England, in 1742, and also the typhus fever in some parts of Massachusetts, as at Holliston, where Mr. Stone, a clergyman, and 14 of his congregation died of it in a short time. In the summer of 1743, when the city of New York contained but 7000 inhabitants, the yellow fever swept off large numbers. The reports of interments show, that from July 25th to September 25, 51 children, and 114 adults were buried, and from September 25 to October 22, 16 children and 36 adults, making 217, a majority of whom perished of this disease. The same was a sickly year in Boston. In 1745 the yellow fever again prevailed in Charleston and New York city, and the malignant dysentery swept away 70 of the inhabitants of Stremford, Conn., out of a few hundreds, all living in one street.

(Webster, vol. 1, 239). The typhus fever, about this period, laid waste the Indian tribes, in consequence of which, the Senecas removed their quarters two or three times in as many years. The whites trading with them were not affected. (Webster). In 1746, the Mohegan tribe suffered severely by the same disease—about one hundred of the tribe perished between August and cold weather. I have called this typhus fever, although if we are to believe Dr. Tracy's account, the disease had some unusual symptoms. "The patient first complained of pain in the head and back, which was followed by fever—in three or four days the skin turned as yellow as gold, a vomiting of black matter took place, and generally a bleeding at the nose and mouth, which continued till the patient died." (Webster, vol. 1, p. 342).

Albany was visited in the same year (1746) with what Colden calls a "nervous pain," and Dangcup the "yellow fever." It seems that the bodies of some of the patients were yellow, and the crisis was on the ninth day. The disease left many in a state of imbecility of mind, approaching to idiocy; others were afterwards troubled with swelled limbs. The disease begun in August, was checked only by frost, and carried off 45 persons. It was believed to have been imported. It was probably yellow fever. The same year a fleet arrived in our waters, under the Duke D'Auville, on board of which a malignant fever prevailed, and one-third of the Indians who visited the cantonments of the soldiers, who landed from the fleet, died. A fever of a fatal kind prevailed at the same time among the Mohegan Indians. In 1737 another epidemic influenza swept over the country, and the yellow fever, or a high grade of bilious, prevailed in Philadelphia; the next year it was fatal in Charleston, S. C.

The dysentery and "Cong. nervous fever," (typhoid fever?) visited many towns in Connecticut, with great mortality. In Waterbury 130 died of dysentery, chiefly. Twenty died of fever in the high mountainous town of Coonwall. Intermittents ravaged Hartford (Conn.) for the last time. (Webster). The measles were common in New England at this time. In 1750 a very fatal fever carried off forty of the inhabitants of Bethlem, Conn., which was attributed to draining a swamp. In 1751, the dysentery was very fatal in New Haven and Hartford, Conn., as well as other places, and maligant angina was a not uncommon disease through New England. In December, 1753, forty-three citizens of Holliston, Mass., were swept off in a short time by an unusual disease, probably typhoid pneumonia. We are told that "the disease begun with a violent pain in the breast, or side, not often in the head; then succeeded a high

7O4

fever, but without delirium. The critical days were the 3d, 4th, 5th, or 6th. Some of the patients appeared to be strangled to death." The town contained but eighty families. The angina maligna was very rife in 1755, and Webster relates that in one town on Long Island, only two children under 12 years of age, survived. In 1757, the influenza again prevailed over North America, and the next year appeared in Europe. In 1758, and 1759, dysentery and measles were both very fatal throughout the country. Typhus fever continued to appear in different places from time to time as in Bethlehem, Conn., in November, 1760, carrying off 40 of the inhabitants in the course of the winter. The disease is represented as having been very violent, often terminating on the third or fourth day, and in some cases fatally within twenty-four hours. This fever seems to have been typhus, or, perhaps, a malignant pleurisy, and prevailed in many parts of Connecticut, as in East Haven, where 45 men died, mostly heads of families—also at New Haven, among the citizens and students of the college, according to Dr. Trumbull, of North Haven, as quoted by Webster, (vol. 1, p. 249, Epid.) "the blood was very thick and sizy—often issuing from the nose, and sometimes from the eyes. The inflammation was violent, and soon produced delirium. *The most robust bodies were most liable to the disease.* A free use of the lancet, in the early stages of the disorder, was the only effectual remedy. Where the physicians were afraid to bleed, the patients all died. This malady prevailed from Nov., 1760 to March, 1761."

Noah Webster, writing in 1789, says: "I cannot learn that this species of inflammatory fever has ever been epidemic in the northern parts of America, since this period. But it is a common winter fever in the Carolinas, after sickly summers; and in the northern States sporadic cases of it occur with all its formidable symptoms. It is the pestilence of winter, and rarely, if ever appears, except when pestilential epidemics are current in summer." (On Epid. vol. 1, p. 249. See Med. Repos., vol. 11, art. by H. Williamson).

